# Carcass characteristics and meat quality of purebred Pakchong 5 and crossbred pigs sired by Pakchong 5 or Duroc boar

**DOI:** 10.5713/ajas.18.0279

**Published:** 2018-09-13

**Authors:** Rachakris Lertpatarakomol, Chanporn Chaosap, Kamon Chaweewan, Ronachai Sitthigripong, Rutcharin Limsupavanich

**Affiliations:** 1 Department of Animal Production Technology and Fisheries, Faculty of Agricultural Technology, King Mongkut’s Institute of Technology Ladkrabang, Bangkok 10520, Thailand; 2 Faculty of Veterinary Medicine, Mahanakorn University of Technology, Bangkok 10530, Thailand; 3 Department of Agricultural Education, Faculty of Industrial Education and Technology, King Mongkut’s Institute of Technology Ladkrabang, Bangkok 10520, Thailand; 4 Bureau of Animal Husbandry and Genetic Improvement, Department of Livestock Development, Bangkok, 10400, Thailand

**Keywords:** Pakchong 5, Terminal Boar, Crossbred Pig, Carcass Characteristic, Meat Quality, Duroc

## Abstract

**Objective:**

This study investigated carcass characteristics and meat quality of purebred Pakchong 5, crossbred pigs sired by Pakchong 5, and crossbred pigs sired by Duroc.

**Methods:**

Forty-eight pigs (average body weight of 22.25 kg) were composed of three groups as purebred Pakchong 5 (PP), Large White×Landrace pigs sired by Pakchong 5 (LWLRP), and Large White×Landrace pigs sired by Duroc (LWLRD). Each group consisted of eight gilts and eight barrows. At 109-day-raising period, pigs were slaughtered, and carcass characteristics were evaluated. *Longissimus thoracis* (LT) muscles from left side of carcasses were evaluated for meat quality and chemical composition. Data were analyzed using general linear model procedure, where group, sex, and their interaction were included in the model.

**Results:**

The PP had greater carcass, total lean, and ham percentages than crossbred pigs (p< 0.05). LWLRP had thicker backfat and more carcass fat percentage than LWLRD (p<0.05). There were no differences (p>0.05) on cutting percentages from tender loin, loin, boston butt, and picnic shoulder among groups. The PP and LWLRP had larger loin eye area (LEA) than LWLRD (p<0.05). Gilts had more loin percentage and lower L* value than barrows (p<0.05). No meat color parameters (L*, a*, and b*) were affected by groups (p>0.05). PP and LWLRP had larger muscle fiber diameters than LWLRD (p<0.05). However, water holding capacity, Warner-Bratzler shear force values, and chemical composition of LT were not affected by group or sex (p>0.05).

**Conclusion:**

Pakchong 5 purebred has good carcass and lean percentages. Compared to Duroc crossbred pigs, Pakchong 5 crossbreds have similar carcass and lean percentages, larger LEA, and slightly more carcass fat, with comparable meat quality and chemical composition. Pakchong 5 boars are more affordable for very small- to medium-scale pig producers.

## INTRODUCTION

Pork is the most important red meat consumed in Thailand with an increased consumption of about 4.18% in 2016 compared to that in 2015 [1]. Similar to other countries, an increasing in lean to fat ratio of pig carcasses has been set as one of the major goals in Thai swine production industry, while meat quality becomes more important. Pig breed particularly is an important factor for improving pork economical traits [2,3], especially using terminal sire or prominent boar [4,5]. Three-line crossbred pigs have intermediate values of parents for carcass and meat quality traits [5,6]. However, pork quality variation among different genders has been reported [2,4,7,8].

In Thailand, Duroc breed is widely used as terminal boar to produce three-line crossbred pigs (Large White×Landrace×Duroc). The synthetic commercial breeds, offered by large-scale pork producers, have become popular terminal boars for the swine production industry [9]. Although 95% of total swine production is for local consumption and majorly supplied by large-scale pork producers [10]. It has been reported in 2015 that the major pork producers, approximately 94.98%, are very small-scale. In addition, 3.68% are small-scale and 1.20% are medium-scale. The prominent boars from commercial breeders are usually unaffordable for the very small- to medium-scale producers. To support these small holders, pig breeds with improved carcass and meat quality traits should be developed for use as terminal sires. Furthermore, these animals should be made economically available to small producers, especially to those in provincial areas.

Pakchong 5 pig has been developed by the Department of Livestock Development of Thailand. It is a synthetic terminal boar established from a genetic combination of 62.5% Duroc and 37.5% Pietrain breeds. It was derived from the *inter se* mating and selected for important economical traits for five generations. These traits include average daily gain of more than 850 g/d, feed conversion ratio of less than 2.50, backfat thickness of less than 1.0 cm, and loin eye area (LEA) of larger than 37 cm^2^ [9]. It was proposed to be used as a terminal boar for producing fast growing and lean fattening pigs for very small- to medium-scale pork holders [9]. Its carcass and meat quality, however, have not been examined. As a result, we investigated carcass characteristics and meat quality of different fattening pigs including purebred Pakchong 5, crossbred pigs sired by Pakchong 5 boar, and crossbred pigs sired by Duroc boar as well as to compare gender effect.

## MATERIALS AND METHODS

### Animal care and use

The experimental procedure on animal care and use was approved by the Institutional Animal Care and Use Committee of King Mongkut’s Institute of Technology Ladkrabang (AGRI-KMITL002/2015).

### Animals and sampling

Forty-eight pigs were included in this study. They were separated into three experimental groups consisting of purebred Pakchong 5 (PP), Large White×Landrace pigs sired by Pakchong 5 boar (LWLRP), and Large White×Landrace pigs sired by Duroc boar (LWLRD). Each group included 16 pigs, which were eight gilts and eight barrows. All pigs were raised in the same fattening pig farm in Nakorn Ratchasima, Thailand. Sixteen animals of each treatment were allocated into a 4×4-m^2^ pen. The pens were constructed with 30-cm-high concrete walls topped with metal railings for well ventilation and non-slip cement flooring. Pigs had access to a shallow pond, which was built inside at the end of each pen, to allow the pigs to clean their bodies and reduce body temperature. Water in the shallow ponds was released into a waste water pond every 3 to 5 days for agricultural utilization or biogas production. Each pen was also equipped with automatic feeders and water dispensers. Feedings were given to the animals *ad libitum* with 2 basal diet formulas for growers (20 to 50 kg body weight [BW]) and finishers (50 to 110 kg BW) as presented in Table 1. Raising period was started when the pigs had average BW of 22.25±3.27 kg and finished at 109 days. No statistical analysis was performed to compare growing characteristics and feed intakes of the experimental treatments. For 109 days of raising period, accumulate feed consumptions of PP, LWLRP, and LWLRD were 297.50, 286.56, and 286.56 kg, respectively. At the end of the experiment, sixteen pigs, with approximate live weight of 100 kg, from each group were fasted for 12 h and transported approximately 15 km or within 30 min driving to the abattoir. Animals were rested for 2 h before slaughtering with free access to water. Each animal was weighed prior to being electrically stunned, bled, scalded, de-haired, eviscerated, and split to obtain carcass data. For meat quality evaluation, *Longissimus thoracis* (LT) muscles from the right side of all carcasses were collected and transported (3°C±2°C) to meat science and technology laboratory, Faculty of Agricultural Technology, King Mongkut’s Institute of Technology Ladkrabang (KMITL), Thailand and kept in a walk-in chiller (2°C±2°C).

### Carcass characteristics

Carcass characteristics were determined from the left side of all carcasses based on hot carcass weight. Carcass length was measured from the cranial edge of the first rib to cranial tip of the aitch bone. Average backfat thickness was measured from the first rib, last rib, and last lumbar. The LEA was determined by tracing on a transparent plastic sheet placed on the cut surface of *Longissimus* muscle between the 10th-11th ribs. The traced LEA was measured using a leaf area meter (LI-3100C, LI-COR Inc. Lincoln, NE, USA). At 45 min postmortem, *Longissimus* muscle pH was directly measured on the loin eye surface on the 10th rib using a pH meter equipped with a spear tip glass electrode (Model SG2 - ELK Seven Go, Mettler-Toledo International Inc., Giessen, Germany). Tender loin and primal cuts, including loin, boston shoulder, picnic shoulder, ham, and belly, were separated from each carcass and weighed. Total lean percentage was calculated from the weights of tender loin and four primal cuts.

### Meat quality

At 24 h postmortem, LT muscle pH was measured using a pH meter equipped with a spear tip glass electrode (Model SG2 - ELK Seven Go, Mettler-Toledo International Inc., Germany). Commission Internationale de I’Eclairage (CIE) L*, a* and b* color values were determined on the cut surfaces of 3-cm-thick LT slices, after allowing to bloom for 45 min at 25°C±2°C, using a portable spectrophotometer (MiniScan EZ, Illuminant D65, 10° standard observer, 2.5-cm-aperture, Hunter Associates Laboratory Inc., Reston, VA, USA). Water holding capacity (WHC) was determined by the method in the literature [5,11]. For drip loss, two pieces of 1.5-cm-thick slices obtained from each LT muscle were weighed, hanged in a closed high-density polyethylene bag and stored at 2°C±2°C for 48 h. Care was taken to protect meat from touching the bag. Drip loss percentage was calculated by measuring weight loss as a percentage of original meat weight. For cooking loss, two pieces of 3-cm-thick slices obtained from each LT muscle were weighed and placed into high-density polyethylene bag. Care was taken to remove air inside the bag as much as possible before heat sealing the package. The sealed package was then placed into a water bath set at 80°C for 40 min or until core temperature of meat sample reached 70°C. Samples were cooled down to room temperature before weighing and calculating sample weight difference before and after cooking as a percentage of weight before cooking. Warner-Bratzler shear force (WBSF) values were derived from using ten 1×1×3 cm^3^ cooked pieces of each cooking loss sample. WBSF was measured using an Instron Universal Testing Machine (model 2519-104, Instron, Norwood, MA, USA) equipped with a 50 kg load cell using 200 mm/min crosshead speed [11]. Muscle fiber diameter was determined from 200 muscle fibers of 100 g samples by using 4× compound microscope equipped with Dino-Eye eyepiece camera to capture images and subsequently evaluated with Dino Capture version 2.0 software (AnMo Electronics Corporation, New Taipei City, Taiwan) [12]. Sarcomere length data were determined from two 2×2×2-cm^3^ cubes of muscle samples according to previous study [13]. Sarcomere length of each muscle sample was calculated from 30 sarcomeres of 30 different muscle fibers. The meat samples were analyzed for dry matter (DM), crude protein (CP), ether extract (EE), and ash by AOAC methods [14]. DM was determined by drying the samples at 105°C in a hot-air oven. CP and EE were determined by the Kjeldahl method and Soxhlet extraction method. Ash was determined at 550°C overnight in a furnace. The soluble and insoluble collagen contents in muscle were determined according to the method described by Hill [15] with slight modifications. The sample was homogenized with Ringer’s solution at 77°C for 70 min and centrifuged for 10 min at 2,500 g. The supernatant solutions were hydrolyzed in 12 N HCl and the sediments were hydrolyzed in 6 N HCl for 24 h at 110°C. The amount of hydroxyproline was calculated from standard curve of hydroxyproline at the absorbance of 550 nm.

### Statistical analysis

Data were considered as a 3×2 factorial arrangement in completely randomized design, with 3 groups (PP, LWLRP, and LWLRD) and 2 sexes (gilt and barrow). The general linear model procedure of SPSS version 17.0 [16] was used for analysis of variance to analyze all parameters included the effects of group, sex, and their interaction. Due to the slaughter weight significantly differing between animals, live weight was fitted as a linear covariate for analyzing carcass characteristic traits. Duncan’s multiple range test was used to compare differences between mean values at a 5% level of significance. For the interaction, mean comparisons were performed among the 6 treatment combinations. If the interaction was not significant, mean comparisons were performed only for main effects.

## RESULTS AND DISCUSSION

### Carcass characteristics

Carcass characteristics of Pakchong 5 purebred and the two groups of crossbred pigs are shown in Table 2. No combined effects of group and sex were found on any carcass traits. Therefore, the influences from main effects are presented. At 109-day-raising period, PP had the lowest live weight compared to the two groups of crossbred pigs (p<0.05). Crossbred pigs were reported to grow more rapidly than purebred pigs, due to the heterosis effect on crossbreds, which had a genetic variation among breeds of sire and dam [17]. Duroc-sired progeny have been reported to have faster BW gain, whereas Pietrain-sired progeny grew more slowly with less fat accumulation [18]. The slower growth rate characteristic of Pietrain, however, was not observed in LWLRP. By 109-day-raising, LWLRP (105.28 kg) was heavier (p>0.05) than LWLRD (101.67 kg), although statistically insignificant. There was no difference on hot carcass weight among treatments (p>0.05). The carcass length of LWLRP was like PP, but it tended (p = 0.08) to be slightly shorter than LWLRD (84.7, 84.7, and 87.7 cm, respectively). PP had greater carcass and total lean percentages than those of the crossbred pigs (p<0.05), but no difference was found between the two crossbred groups (p>0.05). These assured the lean characteristic of PP synthetic breed as purposed [9]. On the other hand, LWLRP had slightly thicker backfat and higher total carcass fat percentage than PP and LWLRD (p< 0.05). But there was no difference (p>0.05) in both traits between PP and LWLRD. Although it is generally acknowledged that the Duroc breed tends to grow faster and accumulate more fat [18]. The commercial Duroc sire for producing LWLRD in this study might be selected for lean production. But the genetic potential of Duroc in Pakchong 5 sire was selected for growth and lean. In the present study, the same feed formulation was offered to the pigs for the whole finishing period without adjustment. As LWLRP rapidly grew to a certain weight, it could likely accumulate more fat. The average backfat thickness of LWLRP, however, was less than 2.5 cm.

For carcass cutting percentages (Table 2), there were no differences on tender loins, loins, boston butts, and picnic shoulders among groups (p>0.05). PP had greater (p<0.05) ham percentage than LWLRP and LWLRD, whereas no difference (p>0.05) was found between the two crossbred pigs. It might be due to purebred Pakchong 5 pigs had the genetic potential from the combination of Pietrain breed. In agreement with researchers who indicated that lean meat and ham percentages of Pietrain breed were higher than those of Duroc breed [19,20]. Belly percentage of PP and LWLRD was similar (p>0.05), but both groups had less belly percentage than LWLRP (p<0.05). The larger proportion of belly in LWLRP was likely due to slightly more carcass fat accumulation compared to the others. On the other hand, LEA of LWLRP did not differ from PP (p>0.05), while both were larger (p<0.05) than that of LWLRD. LEA is generally well correlated to muscle percentage [21]. In contrast to our study, previous research reported that Duroc crossbred (Duroc×Large White) and Pietrain crossbred (Pietrain×Large White) had no statistically significant difference in LEA [22]. No gender effect (p>0.05) was observed on any carcass traits, with the exception for loin percentage. Gilts appeared to have more (p<0.05) loin percentage than barrows. Previous report showed that the proportion of trimmed loin was greater for gilts than that from barrows [2].

### Meat quality

No interaction effects of group and sex were observed on meat quality traits (p>0.05, Table 3). Therefore, the influences of main effects are presented. The LT muscle pH was not affected by group or sex main effect (p>0.05). The values of pH at 45 min and 24 h postmortem of the loin muscles were in normal ranges, as they were previously reported to be 6.4 and 5.6 to 5.8, respectively [23,24]. Therefore, the incidence of pale, soft, and exudative (PSE) and dark, firm, and dry (DFD) pork were not found in the present study.

No meat color parameters (CIE L*, a*, and b* values) were influenced by group (p>0.05). In agreement with several researchers who reported no effect of breed on L*, a*, and b* values of pork loins [4,22]. However, LT muscles from barrows were lighter (higher L*) than those of gilts (p<0.05). This could possibly be due to the difference in muscle fiber type proportion between gilts and barrows. Research has been reported that gilts had higher proportion of type IIb fiber, but lower proportions of type I and IIa fibers than barrows [25]. In general, type IIb fibers are lighter, while type I and IIa fibers are darker and redder [26]. Muscle fiber type I, slow-twitch-oxidative, is small and slow contraction. It has high mitochondria, myoglobin, and lipid contents. Muscle fiber type IIb, fast-twitch-oxidative, is large and fast contraction. It has high glycogen contents [27]. Meta-analysis of gender effects in combination with carcass weight and breed on pork quality found a significant difference among genders for lightness values [8]. They indicated that immunocastrated male pork has the lightest meat color. The same study stated that regarding to pork quality, castrated pigs statistically segregated from the others regardless of type of castration. However, there are controversial in the effect of gender on meat color. Several researchers found no difference in lightness values between genders [4,7].

PP and LWLRP had a larger muscle fiber diameter than LWLRD (p<0.05, Table 3). Muscle fiber cross-sectional area was also reported to be positively correlated to LEA [28]. This may explain an earlier result of larger LEA found in PP and LWLRP, when compared to that of LWLRD (Table 2). Previous research studied in different pig breeds and reported that myosin heavy chain IIb fibers are the most abundant in pigs having large LEA [29]. The authors suggested that myosin heavy chain IIb fiber is the determining fiber contributing to the differentiation of large and small LEA in the pig. Although, previous study indicated that larger muscle fiber diameter may result in reduced tenderness and WHC of pork loin [30]. We found no differences in WBSF and WHC (drip loss and cooking loss) among groups (p>0.05).

There was no significant difference in sarcomere length among groups (p>0.05). Loin muscles from barrows, however, had longer sarcomere length than those from gilts (p<0.05). Sarcomere length has been associated with meat tenderness [13]. But we found no significant difference in WBSF values between sexes (p>0.05). The difference in sarcomere length could be related to the type of muscle fibers dominant in the muscle. Type IIb fibers have been characterized by a shorter sarcomere length than type I fibers [31].

The chemical composition of LT muscles was not affected by group or sex main effect (p>0.05, Table 4). Similarly, no influence of crossbreeding or sex was found on chemical composition of pork loins from Duroc×Large White crossbreds and Pietrain×Large White crossbreds [7]. Although statistically insignificant, LWLRP had slightly more (p>0.05) fat content in the loin muscle than LWLRD (2.11% and 1.84%, respectively). Intramuscular fat positively influences sensory quality traits of meat including flavor, juiciness, and tenderness [32]. A minimum intramuscular fat content of 2.2% to 3.4% was recommended to improve eating acceptability of pork [33]. Furthermore, no influence of group or sex main effect was observed on collagen content of pork loins (p>0.05). This is expected as all pigs were slaughtered at the same age. No significant difference on collagen contents among pig breeds with the same age has been reported [34]. Previous study suggested that as an animal ages, the proportion of mature intermolecular crosslinks in collagen molecules increases, resulting in meat toughness [35].

## CONCLUSION

With lighter BW, Pakchong 5 purebred provides good carcass and lean percentages. Comparing to commercial Duroc crossbred pigs, Pakchong 5 crossbred pigs have similar carcass and lean percentages, a larger LEA, and slightly more carcass fat, with similar meat quality and chemical composition. Feed formulation during finishing may be adjusted to accommodate appropriate fat deposition. Gilts have more loin percentage and darker loin color than barrows, which might be due to muscle fiber type proportion variation. Since developed by governmental agency, Pakchong 5 terminal boars are more affordable for very small- to medium-scale pig producers. The sensory quality and consumer acceptance of meat from Pakchong 5 crossbred pigs are to be further investigated.

## Figures and Tables

**Table 1:** Formula of basal diets

Ingredients (%)	Grower (20–50 kg BW)	Finisher (50–110 kg BW)
Broken rice	51.70	54.86
Rice bran	15.00	19.95
Soybean meal	21.00	15.96
Fish meal	5.00	3.99
Di-calcium phosphate	4.00	3.99
Vegetable oil	2.00	0.00
Salt	0.50	0.50
Antibiotic	0.30	0.25
Premix1)	0.50	0.50
Calculated analysis		
ME (kcal/kg)	3,214	3,156
Crude protein (%)	17.98	16.08

BW, body weight; ME, metabolizable energy.

1) Supplied per kg diet: Vitamin A 8,000,000 IU; Vitamin D 1,500,000 IU; Vitamin E 40,000 ppm; Vitamin K 1,500 ppm; Thiamin 1,000 ppm; Riboflavin 4,000 ppm; Vitamin B_12_ 20 ppb; Pyridoxine 2,000 ppm; Niacin 20,000 ppm; Biotin 30 ppm; Folic acid 600 ppm; Se 250 mg; I 200 mg; Fe 60,000 mg; Mn 25,000 mg; Zn 60,000 mg; Cu 15,000 mg.

**Table 2:** Means±standard deviation of carcass traits of finished Pakchong 5 pig and crossbred pigs sired by Pakchong 5 or Duroc boar

Items	Group (G)1)			Sex (S)		p-value		
		
	PP	LWLRP	LWLRD	Gilt	Barrow	G	S	G×S
Live weight (kg)	93.56±6.54^b^	105.28±8.47^a^	101.67±7.90^a^	98.53±8.15	101.81±9.77	<0.01	0.13	0.09
Hot carcass weight (kg)	84.49±6.01	83.76±6.87	83.15±6.83	83.50±6.69	84.09±8.10	0.12	0.22	0.83
Carcass length (cm)	84.69±4.90	84.74±3.44	87.69±3.70	85.83±4.93	85.58±4.38	0.08	0.83	0.88
Carcass percentage	79.89±1.51^a^	78.1±2.14^b^	78.32±0.99^b^	78.62±1.11	78.93±2.17	0.01	0.51	0.74
Backfat thickness (mm)	19.36±3.67^b^	22.27±5.24^a^	18.84±4.16^b^	19.32±3.53	20.99±5.59	0.03	0.14	0.95
Total lean percentage2)	47.04±2.38^a^	44.36±1.77^b^	45.03±1.53^b^	45.90±2.36	45.05±1.99	<0.01	0.15	0.46
Total fat percentage	11.56±1.97^b^	12.94±2.27^a^	11.33±1.09^b^	11.49±1.49	12.39±2.27	0.02	0.08	0.10
Cutting percentage								
Tender loin	1.41±0.14	1.36±0.17	1.36±0.13	1.38±0.14	1.38±0.16	0.63	0.93	0.87
Loin	8.33±0.68	8.16±0.86	8.22±0.73	8.52±0.74^x^	7.95±0.68^y^	0.83	0.01	0.62
Boston butt	4.73±0.53	4.60±0.39	4.41±0.28	4.58±0.40	4.57±0.45	0.18	0.93	0.08
Picnic shoulder	10.35±0.85	10.34±1.11	10.47±0.88	10.49±0.95	10.28±0.93	0.93	0.49	0.48
Ham	21.42±1.37^a^	19.37±1.46^b^	19.89±1.07^b^	20.33±1.76	20.12±1.33	<0.01	0.60	0.29
Belly	12.42±1.14^b^	13.39±1.47^a^	12.53±1.07^b^	12.57±1.05	12.99±1.32	0.04	0.23	0.73
Loin eye area (cm^2^)	55.12±9.47^a^	53.62±9.70^a^	47.21±7.62^b^	53.24±10.55	50.73±8.35	0.04	0.34	0.11

1) PP, purebred Pakchong 5; LWLRP, Large White×Landrace pigs sired by Pakchong 5 boar; LWLRD, Large White×Landrace pigs sired by Duroc boar.

2) Total lean percentage was calculated from tender loin, loin, boston butt, picnic shoulder, and ham.

Means within the same row with different superscript letters (^a,b^) under group effect differed at p<0.05. Means within the same row with different superscript letters (^x,y^) under sex effect differed at p<0.05.

**Table 3:** Means±standard deviation of meat quality parameters of *Longissimus* muscle from finished Pakchong 5 pig and crossbred pigs sired by Pakchong 5 or Duroc boar

Items	Group (G)1)			Sex (S)		p-value		
		
	PP	LWLRP	LWLRD	Gilt	Barrow	G	S	G×S
pH_45min_	6.13±0.26	6.19±0.28	6.34±0.28	6.25±0.30	6.18±0.28	0.08	0.39	0.34
pH_24h_	5.75±1.54	5.71±0.13	5.76±0.10	5.76±1.37	5.71±0.09	0.58	0.12	0.67
Meat color								
L* (lightness)	52.42±2.47	52.12±2.74	51.19±3.18	50.67±2.40^y^	53.16±2.90^x^	0.39	<0.01	0.87
a* (redness)	3.28±1.30	3.96±1.17	3.41±1.00	3.81±1.09	3.29±1.17	0.22	0.12	0.19
b* (yellowness)	11.82±1.07	12.32±1.09	11.47±0.81	11.67±1.07	12.08±1.01	0.06	0.16	0.38
Water holding capacity (%)								
Drip loss	3.16±0.72	3.11±0.67	3.15±0.78	3.23±0.83	3.05±0.58	0.98	0.41	0.85
Cooking loss	22.10±3.25	21.75±3.08	22.50±2.53	22.49±2.84	21.75±2.77	0.78	0.40	0.34
Muscle fiber diameter (μm)	71.27±5.81^a^	71.71±4.27^a^	65.70±6.53^b^	70.15±6.91	68.97±5.40	0.01	0.51	0.61
Sarcomere length (μm)	1.71±0.14	1.63±0.06	1.66±0.10	1.63±0.08^y^	1.70±0.13^x^	0.10	0.02	0.40
Shear force value (kg)	6.29±0.69	6.49±0.79	6.28±1.19	6.50±1.00	6.20±0.82	0.77	0.27	0.58

1) PP, purebred Pakchong 5; LWLRP, Large White×Landrace pigs sired by Pakchong 5 boar; LWLRD, Large White×Landrace pigs sired by Duroc boar.

Means within the same row with different superscript letters (^a,b^) under group effect differed at p<0.05. Means within the same row with different superscript letters (^x,y^) under sex effect differed at p<0.05.

**Table 4:** Means±standard deviation of chemical composition and collagen contents of *Longissimus* muscle from finished Pakchong 5 pig and crossbred pigs sired by Pakchong 5 or Duroc boar

Items	Group (G)1)			Sex (S)		p-value		
		
	PP	LWLRP	LWLRD	Gilt	Barrow	G	S	G×S
Chemical composition (%)								
Moisture	74.17±0.74	73.28±0.85	73.79±1.21	73.66±0.97	73.83±1.04	0.11	0.61	0.08
Crude protein	23.17±1.06	23.83±1.30	23.58±1.68	23.72±1.59	23.33±1.10	0.55	0.44	0.22
Crude fat	1.51±0.51	2.11±0.76	1.84±0.59	1.82±0.86	1.82±0.40	0.15	0.98	0.79
Ash	1.02±0.12	1.03±0.12	1.02±0.11	1.02±0.11	1.03±0.12	0.96	0.72	0.76
Collagen contents (mg/g)								
Insoluble collagen	3.68±1.12	3.36±0.65	3.46±1.16	3.47±0.90	3.54±1.09	0.76	0.84	0.14
Soluble collagen	0.54±0.15	0.59±0.14	0.49±0.19	0.54±0.15	0.54±0.18	0.28	0.94	0.06
Total collagen	4.23±1.24	3.96±0.77	3.94±1.34	4.00±1.03	4.08±1.22	0.82	0.84	0.13

1) PP, purebred Pakchong 5; LWLRP, Large White×Landrace pigs sired by Pakchong 5 boar; LWLRD, Large White×Landrace pigs sired by Duroc boar.
